# Aβ_42_ regulates ER cholesterol turnover to modulate ER‐microtubule contact tethering and alter Tau dynamics in Alzheimer's disease

**DOI:** 10.1002/alz70855_101226

**Published:** 2025-12-23

**Authors:** Kevin R Shen, George C Shum, Abby C Woods, Tayler B Belton, Eric D Leisten, Yvette C Wong

**Affiliations:** ^1^ Northwestern University, Chicago, IL, USA

## Abstract

**Background:**

Alzheimer's disease (AD) is characterized by the pathological accumulation of both amyloid‐beta (Aβ) and cytoplasmic tau, but their mechanistic crosstalk remains to be further investigated. Moreover, while cholesterol dyshomeostasis has been implicated in AD, its role is still not completely understood. Cholesterol homeostasis is tightly regulated at the endoplasmic reticulum (ER), a highly dynamic and structurally complex organelle, where the enzyme ACAT1 turns over cholesterol into cholesteryl esters. Importantly, the ER also dynamically tethers to microtubules via ER‐microtubules contact sites, and are implicated in dendritic spine stability and memory. However, whether ACAT1 functionally interacts with Aβ to regulate ER cholesterol and ER structure, and its downstream regulation of ER‐microtubule contact sites and tau dynamics have never been studied.

**Method:**

We utilized super‐resolution live Lattice SIM2 microscopy to uncover a new mechanistic pathway connecting cholesterol turnover, Aβ function, and tau aggregation in AD. We examined the role of ER cholesterol in modulating ER ultrastructure and dynamics over time, and a converging role for Aβ production and Aβ42 versus Aβ40 in regulating this pathway. We further conducted *in silico* structural multimer modeling of Aβ42 and Aβ40's structural interactions with ACAT1's catalytic pocket. Finally, we investigated cholesterol's role in modulating ER‐microtubule contact site tethering via STIM1‐EB binding, and its impact on downstream tau microtubule dynamics and aggregation.

**Result:**

We found that accumulation of ER cholesterol resulted in the dynamic formation of ER spheres as a novel structural component of the ER network. Remarkably, inhibition of Aβ generation also induced ER sphere formation, supporting a role for Aβ regulation of ER cholesterol's turnover. Mechanistically, Aβ42 but not Aβ40 structurally interacted with key catalytic residues of ACAT1 to promote ACAT1's turnover of cholesterol, which was supported by reduced ER sphere formation in AD‐associated mutant APP with increased Aβ42 production. Functionally, ER cholesterol resulted in the downstream untethering of ER‐microtubule contact sites mediated by STIM1 and EB, ultimately leading to tau dissociation from microtubules and oligomerization.

**Conclusion:**

Our work provides evidence for a unifying mechanism linking Aβ function with tau dynamics through cholesterol‐mediated ER dynamics, and identifies a novel cellular pathway underlying AD pathogenesis.

